# Lead-Time Corrected Effect on Breast Cancer Survival in Germany by Mode of Detection

**DOI:** 10.3390/cancers16071326

**Published:** 2024-03-28

**Authors:** Laura Schumann, Moritz Hadwiger, Nora Eisemann, Alexander Katalinic

**Affiliations:** 1 Institute of Social Medicine and Epidemiology, University of Luebeck, 23562 Luebeck, Germanynora.eisemann@uksh.de (N.E.); alexander.katalinic@uksh.de (A.K.); 2 Institute of Cancer Epidemiology, University of Luebeck, 23562 Luebeck, Germany

**Keywords:** mammography, screening, survival, detection mode, lead time

## Abstract

**Simple Summary:**

An important component in the study of survival in breast cancer patients is how the tumor is detected. This involves examining whether and to what extent survival differs when the tumor is detected, for example, by screening or by symptoms. In such an analysis, it is important to note that survival times are influenced by several biases. One important factor is the bias due to the so-called lead time, i.e., the time by which the diagnosis has been brought forward by screening. Therefore, survival may appear to be longer even if earlier diagnosis did not affect disease progression. We examine whether there is a remaining survival difference after correction for bias that could be attributable to the mode of detection, for example, because of higher quality of care. These results can then be used to further improve the survival of breast cancer patients.

**Abstract:**

(1) Background: Screen-detected breast cancer patients tend to have better survival than patients diagnosed with symptomatic cancer. The main driver of improved survival in screen-detected cancer is detection at earlier stage. An important bias is introduced by lead time, i.e., the time span by which the diagnosis has been advanced by screening. We examine whether there is a remaining survival difference that could be attributable to mode of detection, for example, because of higher quality of care. (2) Methods: Women with a breast cancer (BC) diagnosis in 2000–2022 were included from a population-based cancer registry from Schleswig-Holstein, Germany, which also registers the mode of cancer detection. Mammography screening was available from 2005 onwards. We compared the survival for BC detected by screening with symptomatic BC detection using Kaplan–Meier, unadjusted Cox regressions, and Cox regressions adjusted for age, grading, and UICC stage. Correction for lead time bias was carried out by assuming an exponential distribution of the period during which the tumor is asymptomatic but screen-detectable (sojourn time). We used a common estimate and two recently published estimates of sojourn times. (3) Results: The analysis included 32,169 women. Survival for symptomatic BC was lower than for screen-detected BC (hazard ratio (HR): 0.23, 95% confidence interval (CI): 0.21–0.25). Adjustment for prognostic factors and lead time bias with the commonly used sojourn time resulted in an HR of 0.84 (CI: 0.75–0.94). Using different sojourn times resulted in an HR of 0.73 to 0.90. (4) Conclusions: Survival for symptomatic BC was only one quarter of screen-detected tumors, which is obviously biased. After adjustment for lead-time bias and prognostic variables, including UICC stage, survival was 27% to 10% better for screen-detected BC, which might be attributed to BC screening. Although this result fits quite well with published results for other countries with BC screening, further sources for residual confounding (e.g., self-selection) cannot be ruled out.

## 1. Introduction

Breast cancer is the most common cancer among women in Germany. In 2020, there were 70,550 new cases, an additional 6000 in situ tumours, and roughly 18,400 deaths from invasive breast cancer in women [1]. Survival depends, among other factors, substantially on the stage of breast cancer at diagnosis [2,3].

Mammography screening programmes aim to detect breast cancer at an early stage. Based on European guidelines, the German National Mammography Screening Programme was implemented throughout Germany between 2005 and 2009 [4]. Women aged between 50 and 69 years are invited for screening every two years. Participation is voluntary and there are no out-of-pocket costs. Screening takes place in certified screening units [4,5].

A number of studies have described the association between the mode of cancer detection and survival [6,7]. The tumour characteristics of breast cancers detected by mammography screening are more favourable than those detected outside the screening programme, i.e., they are of lower stage and lower grade [8,9,10,11]. The observed survival of patients with screen-detected cancers is better than that of patients with cancers detected outside screening programmes [9,12,13,14].

However, the observed survival difference by mode of breast cancer detection is likely influenced by lead time bias. Lead time bias only affects cases detected through systematic screening or other early detection. Survival estimates for screen-detected cancers may be inflated because these patients are diagnosed earlier than the onset of symptoms. As a result, survival may appear longer even if the earlier diagnosis had no effect on disease progression [7,12,15]. Therefore, the lead time must be subtracted in order to make valid comparisons between groups with and without screening [16]. There are several studies that account for lead time bias with the method described by Duffy et al. [17]. This method is based on the so-called sojourn time. The sojourn time is the time duration when the tumour is asymptomatic but can be detected by screening.

The aim of this study was to investigate the long-term survival under contemporary screening settings of women after cancer diagnosis in Schleswig-Holstein, Germany, stratified by mode of cancer diagnosis.

## 2. Materials and Methods

### 2.1. Data Source

Anonymized data were provided by the population-based Cancer Registry of Schleswig-Holstein, which is the northern most federal state in Germany. The registry was established in 1997 and covers a population of 2.9 million inhabitants. Physicians in Schleswig-Holstein are legally obliged to report cancer cases to the cancer registry. The completeness of the cancer registry has been over 95% in recent years [18,19]. It contains information on age, sex, tumor characteristics, date of diagnosis, vital status, and mode of detection.

### 2.2. Study Population

We included women with breast cancer (ICD-code C50 or D05) diagnosed between 2000 and 2022 (*n* = 68,517). We excluded cases reported only by death certificates (*n* = 5162). For analyses, we considered only patients in the screening age group (50–69 years at diagnosis). Patients who were younger or older were excluded (*n* = 31,186).

The mode of breast cancer detection is recorded by the cancer registry through reports from the treating physician. There were five groups of modes of cancer detection: (1) symptomatic cancer, (2) mammography screening, (3) (other) early detection, (4) self-examination, and (5) missing. If more than one mode of detection was recorded for a case, the following hierarchy was used to assign a specific mode of cancer detection to each case: symptomatic cancer took precedence over all other modes, followed by mammography screening and early detection.

### 2.3. Statistical Analysis

The outcome was all-cause death between the breast cancer diagnosis and 31 April 2022. Vital status was established for each case by regular linkage of cancer registry data with the official registration office. We calculated the 15-year survival after a breast cancer diagnosis.

We present descriptive statistics of tumour characteristics based on the five different modes of breast cancer detection. For the following survival analyses, we excluded patients in whom the mode of detection was missing. Patients who were alive at the end of the observation period were censored. The period between the breast cancer diagnosis and death or censoring was defined as the follow-up time.

A *p*-value of 0.05 was regarded as statistically significant. All statistical analyses were carried out in R 4.1.1 [20]

#### 2.3.1. Survival Analysis

To illustrate survival, we used an analysis of unadjusted survival curves (Kaplan–Meier time-to-event curves) and Cox proportional hazard regressions. The symptomatic cancer group was always the reference group.

First, we presented crude survival curves for all modes of detection and simple Cox proportional hazards regression with the four groups, where the mode of detection was known. Additionally, we performed a multivariable Cox proportional hazard regression adjusting for age, UICC stage and grading. During 2000 and 2022, TNM versions 6, 7 and 8 were in use [21,22,23].

Second, the lead time-corrected survival of the mammography group was then compared to the group with symptomatic cancer in simple and multivariable Cox proportional hazard regression, adjusting for the above-mentioned variables.

The proportional hazard assumption was assessed by visual inspection. No major violations were detected. 

#### 2.3.2. Lead Time Bias Correction

We corrected the survival of the group of patients with mammography screening for lead time bias by subtracting the survival time resulting from lead time bias (“additional survival time”) from the observed survival time. We used Duffy et al.’s (2008) method to estimate the additional survival time for each individual [17]. Two parameters were required for this: the individual follow-up time until censoring or death and a fixed parameter, the sojourn time. The sojourn time is the period of time during which the tumour is asymptomatic but can be detected by screening. It is estimated from clinical studies [17]. Similar to Duffy et al. [17], we assumed that the sojourn time follows an exponential distribution. For the main analysis, we used sojourn times of 3.7 years for the 50–59 age group and 3.9 years for the 60–69 age group, which were suggested by Tabar et al. [24].

We applied the correction solely to the group of patients with mammography screening because (1) this correction is only applicable to tumours in the early detection area, which in our data includes the mammography screening and early detection groups, and (2) the reported sojourn times were derived for mammography screening only. 

#### 2.3.3. Sensitivity Analyses

As a sensitivity analysis, we performed simple and multivariable Cox proportional hazards regressions with lead time bias corrections for two additional and more recently published sojourn time estimations. Aarts et al. [25] presented sojourn times of 4.6 years for age group 50–69, and Choi et al. [26] presented times of 2.5 years for age group 50–59 and 3.1 years for age group 60–69. 

To account for possible uncertainties in the allocation to groups of mode of detection, we performed a second sensitivity analysis comparing the symptomatic cancer group to a pooled group consisting of the mammography screening group, the early detection group, and the self-examination group. For the mammography screening group, we corrected for lead time bias using again the sojourn time by Tabar et al. [24]. We plotted Kaplan–Meier time-to-event curves for the pooled group with and without lead-time bias correction, and fitted simple and multivariable Cox proportional hazard regressions using the same adjustment variables as before. 

## 3. Results

In total, the analyses included 32,169 women in the screening age group (50–69 years) with a breast cancer diagnosis in 2000 to 2022. The cancer was detected due to symptoms in 2334 women, by mammography screening in 9329 women, by other early detection in 4762 women, and by self-examination in 2917 women; in 12,827 women, information on the mode of cancer detection was missing. Characteristics of the study population, stratified by the mode of cancer detection, are illustrated in Table 1. On average, cases with a symptomatic tumour had a higher stage and a higher tumour grading. Thus, two-thirds of all screen-detected tumours had UICC stage 0 or I, while about one-quarter of patients with a symptomatic tumour had stage 0 or I (Table 1). 

The median follow-up time was 8.3 years (interquartile range 3.6 to 13.8 years) and varied only slightly between the five groups. A total of 7160 (22%) deaths could be observed. Concerning the mode of cancer detection, 918 (39%) deaths were observed in the symptomatic tumour group, 883 (9%) deaths in the mammography screening group, 698 (15%) deaths in the early detection group, 797 (27%) deaths in the self-examination group, and in 3864 (30%) deaths the mode of cancer detection was missing.

**Figure 1:**
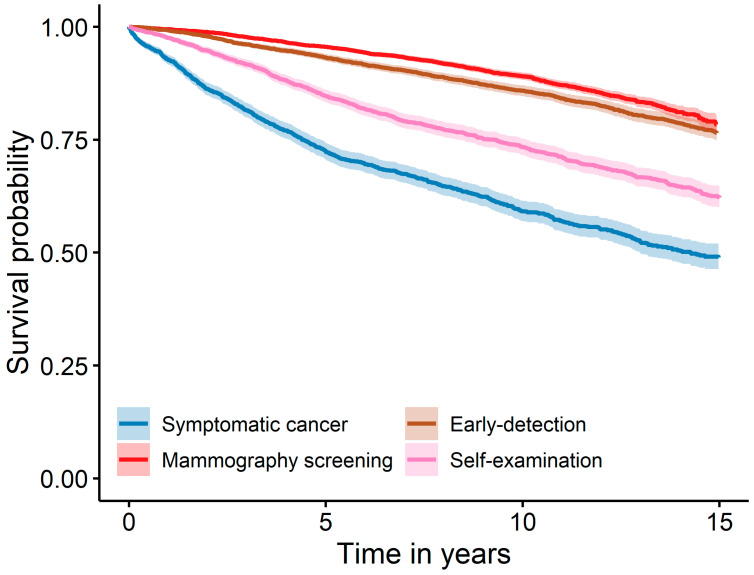
Survival of breast cancer for different modes of detection with the pointwise 95% confidence interval in shaded bands.

In the unadjusted Kaplan–Meier time-to-event curve, patients with a symptomatic tumour had the lowest survival probability compared to the other three groups. The mammography screening group had the highest probability of survival, closely followed by the early detection group (Figure 1).

Accordingly, the hazard for all cause-death in the simple Cox regression was lowest in the mammography screening group (hazard ratio (HR) with the symptomatic tumour group as a reference: 0.23, 95% confidence interval (CI): 0.21; 0.25). Adjusting for UICC stage, tumour grading, and age (in years) reduced the differences between groups (HR: 0.50; 95% CI: 0.45; 0.55) (Table 2).

When comparing patients with symptomatic cancer to patients with screen-detected cancer and correcting the latter group for lead time bias (with the sojourn time by Tabar et al. [24]), a survival advantage of the screen-detected cancer group was observed (HR = 0.36). The survival advantage remained after additionally adjusting for age, UICC stage, and grading, but the estimated survival difference between these two groups decreased to an HR of 0.84 (95% CI: 0.75; 0.94) All results were statistically significant (Table 2).

**Figure 2:**
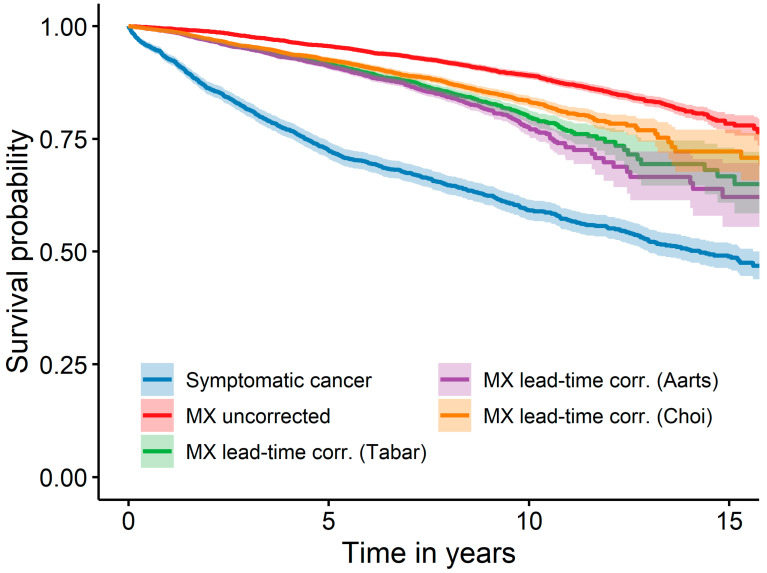
Survival of symptomatic cancer and cancer detected at mammography screening, with the latter corrected for lead time bias additionally with three different sojourn times; Abbreviation: MX—mammography screening.

The first sensitivity analysis compared different sojourn times and revealed that a shorter sojourn time, as published by Choi et al. [26], resulted in larger differences and a longer sojourn time, as published by Aarts et al. [25], which itself resulted in smaller differences in the Kaplan–Meier curve (Figure 2). This effect persists in both simple and multivariable Cox regression (Table 3). The corresponding HR values were significantly different from 1, except the HR in the multivariable Cox regression using the short sojourn time by Aarts et al. [25] (*p* = 0.061) (Table 3).

**Figure 3:**
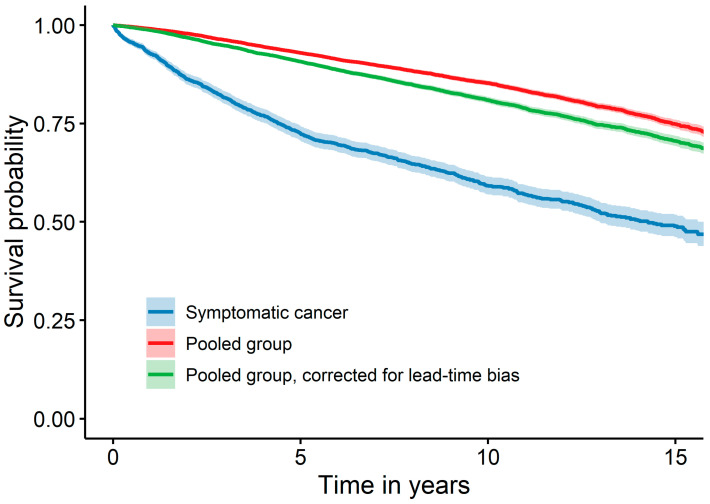
Survival of symptomatic cancer compared to the pooled group consisting of self-examination, early detection and cancer detected at mammography screening, with the latter corrected for lead time bias (with the sojourn time by Tabar et al. [24]) additionally.

The second sensitivity analysis showed a similar result for the pooled group of patients with tumours detected by mammography screening, early detection or self-examination compared with the main analysis, which excluded tumours detected by early detection and self-examination. In the Kaplan–Meier curves, the group of patients with symptomatic cancer had a lower probability of survival than the pooled group. The survival decreased after correction for lead-time bias (with the sojourn time by Tabar et al. [24]), but not below the survival probability of the group symptomatic cancer (Figure 3). The Cox proportional hazard regressions with and without correction for lead time bias indicated similarly robust results. The multivariable model with lead time bias correction yielded an HR that was comparable to the main analysis, but slightly more extreme, namely a HR of 0.71 (95% CI: 0.65; 0.77) (Table 4).

## 4. Discussion

This analysis of 32,169 female breast cancer patients including 7160 all-cause deaths showed that survival varied depending on the mode of breast cancer detection. The characteristics of screening-detected tumours in terms of UICC stage and grading were more favourable than those of symptomatic tumours. We observed a lower survival probability in patients with symptomatic breast cancer compared to patients whose breast cancer was detected by mammography screening (HR: 0.23; CI: 0.21; 0.25). After adjusting for additional variables or correcting for lead-time bias with different sojourn times, or both, the difference in survival was still observed, but the observed difference was smaller (HR ranging between 0.36 and 0.90; Table 2, Table 3 and Table 4).

It is already known that the survival benefit of screening is influenced by the lead time bias, so it is important to have a correction for this bias [15]. A common and often used method to correct for lead time bias is described by Duffy et al. [17]. A parameter, the sojourn time, is needed to calculate the lead time bias-corrected survival time. Most analyses use the sojourn time reported by Tabar et al. in 2000 [6,14,15,27]. With new therapies and changing screening practices, it is important to consider whether the sojourn time needs to be adapted over time and assess if that changes the results. Therefore, we used two more recent sojourn times published by Aarts et al. [25] in 2019 and Choi et al. [26] in 2023. As the sojourn time is estimated using data from a given study population at different observation times, they will differ from each other. One factor that influences the estimation of the sojourn time is the sensitivity. This changed significantly with the introduction of digital mammography in 2002 [25,28]. Furthermore, clinical variables within the study population, such as breast density, can impact sensitivity [26]. Of the sojourn times used in this analysis, the sojourn time of Aarts et al. [25] appears to possess the highest potential for applicability to our study population, as it is based on a Dutch population aged 50–69 years who were invited for screening every two years, as happens in Germany. They also used the period after the introduction of digital mammography to estimate the sojourn time.

The importance of lead time correction is well documented [7,12,15]. The correction method used by Duffy et al. [17] is a widely used and easy-to-implement method. A major advantage is that it can be used for almost any dataset, since only the observed personal survival time and the sojourn time—which can be taken from the literature—are required. In addition, its use is well established in the current literature so that comparisons between different studies and data are possible. However, the methodology has limitations: The assumption of a single sojourn time for all individuals in the study, who have a variety of tumors, seems unrealistic and inaccurate. Abrahamsson et al. [29] developed a new model in which conditional lead time distributions are estimated with information on the tumor size, screening history, mode of detection, and breast density of each individual. The comparison of this new method to the method by Duffy with observational data from Sweden shows that there are only small differences in the correction for the whole analysis. However, differences become visible in subgroup analyses. For small tumors, the differences are still quite small, but for larger tumors, the Duffy et al. [17] method over-corrects, resulting in a shorter estimated survival [29]. However, this new approach was not feasible in our study because we did not have the necessary individual data.

We ran the Cox regressions with these three different sojourn times. The HR ranged between 0.73 for the shortest and 0.9 for the longest sojourn time, and all indicated a survival benefit for the mammography group, although the confidence interval around the 0.90 HR just included the 1 (95% CI: (0.80; 1.01)). This indicates the need to use a sojourn time estimate that was derived from a population and context similar to the study under investigation.

Abrahamsson et al.’s research indicates that our correction method tends to underestimate survival in the mammography screening group, resulting in an overestimation of the HR when we compare the mammography screening group to the symptomatic cancer group. Consequently, the lack of significance in the sensitivity analysis with Aarts et al.’s sojourn time may be due an overcorrection by our methodology [29].

Reviewing the current literature, we identified three possible approaches to accounting for lead-time bias which are applicable to our data. First, regressions are adjusted for variables that are likely to correct for lead time bias, such as tumour size and lymph node status. All these studies concluded that mammography screening is an independent prognostic factor (with an HR ranging between 0.38 and 0.79) [12,13,14]. The second methodological approach was used in the analysis by Lawrence et al. [15]. They corrected for lead time bias using the method described by Duffy [17], without adjusting the model for any other variables. The authors found that screening mammography led to a significant improvement in the survival (HR for outcome survival = 2.5) [15]. Combining the latter two methods resulted in the third approach. These models used Duffy’s lead time bias-corrected survival time and adjusted for additional variables. This approach is the most conservative one, with adjustments applied in two ways: for lead time bias and other potential bias. Allgood et al. concluded that there was still a survival benefit from screening, except when they additionally adjusted for NPI (a validated prognostic tool based on tumour size, grade, and lymph node status) (HR 0.9 vs. 1.2) [6]. A different conclusion was reached by O’Brien et al. When they corrected for both lead time bias and prognostic variables, there was no statistically significant survival benefit, with HR ranging between 0.93 and 1.03. When correcting only for prognostic variables without taking lead time bias into account, a survival benefit was observed (HR between 0.39 and 0.43) [27].

Our results are in line with the other studies that compared survival according to the mode of cancer detection. Symptomatic breast cancers had a higher stage and grading. Survival was better for women whose breast cancer was diagnosed through a screening programme compared with symptomatic cancers [6,7,9,12,13,14,27].

Our adjusted mortality reduction for screening against symptomatic breast cancer detection with the last-named approach ranged between 10% to 27% depending on the sojourn time used (Table 3). These results are in line with a review of RCTs from 2013 that found a reduction of 19% in mortality [30]. A more recent review of incidence-based mortality evaluations from 2020 found a mortality reduction of 22% [31].

There are several limitations to our study. Firstly, self-selection by socioeconomic status could influence the likelihood of screening participation. It is well known that women with lower socioeconomic status have a lower participation rate [32]. It is well established that socio-economic status has an impact on survival rates. Research has demonstrated that there can be up to an 8% difference in survival rates among breast cancer patients based on their residential area in Hamburg [33]. Secondly, we cannot exclude the possibility that doctors coded the mode of cancer detection incorrectly, as there is no official definition. To account for this possibility, we performed a sensitivity analysis combining all screening options into one group. The result was robust. In addition, the incorrect coding was most likely a non-differential misclassification, leading to more conservative estimates. Thirdly, the analysis has the potential for residual confounding because important clinical variables, such as hormone receptors, were missing from the data set. Thus, the observational nature of the study design limits causal conclusions. It should also be mentioned that the coding of tumours has changed over time, as TNM versions 6 to 8 were used in our observation period. The difference is that TNM 6 does not consider micrometastases. Therefore, all tumours with N1 are classified as stage 2. In versions 7 and 8, tumours with N1mi are still classified as stage 1. This leads to a non-differential misclassification, as it is the same misclassification in all groups studied; thus, the effect tends to be underestimated.

A strength of this analysis that our data are up-to-date. The majority of studies examine 5- to 10-year survival [6,12,14,15,27] and only few studies have focused on long-term survival [9,13]. With our data, we were able to calculate 15-year survival to study long-term survival. As the earliest diagnosed cancer was registered in 2000, we can conduct an analysis of long-term survival under the contemporary conditions of screening programmes. Another strength of this analysis is that according to our knowledge, this is the first analysis that compares different sojourn times and how they affect the results.

## 5. Conclusions

Survival varied by mode of detection. Following well-established adjustment, mammography screening is an independent prognostic factor in long-term survival. However, using more up-to-date data for adjustment, the survival advantage becomes smaller. Taken together, our results suggest that the mode of detection is an important factor in long-term survival.

## Figures and Tables

**Table 1:** Patient and tumour characteristics by the mode of breast cancer detection.

Patient and Tumour Characteristics	Mode of Breast Cancer Detection					
	Symptomatic Cancer (*n* = 2334)	Mammography Screening (*n* = 9329)	Early Detection (*n* = 4762)	Self-Examination (*n* = 2917)	Missing (*n* = 12,827)	Overall (*n* = 32,169)
Age in years, mean (SD)	60.0 (5.9)	60.2 (5.9)	59.7 (5.9)	59.7 (5.9)	60.0 (5.6)	60.0 (5.8)
Year of diagnosis						
2000–2004	135 (5.8%)	72 (0.8%)	305 (6.4%)	201 (6.9%)	5968 (46.5%)	6681 (20.8%)
2005–2009	745 (31.9%)	2184 (23.4%)	1585 (33.3%)	1151 (39.5%)	2130 (16.6%)	7795 (24.2%)
2010–2014	839 (35.9%)	3216 (34.5%)	1148 (24.1%)	756 (25.9%)	1274 (9.9%)	7233 (22.5%)
2015–2019	481 (20.6%)	2774 (29.7%)	1202 (25.2%)	557 (19.1%)	2279 (17.8%)	7293 (22.7%)
2020–2022	134 (5.7%)	1083 (11.6%)	522 (11.0%)	252 (8.6%)	1176 (9.2%)	3167 (9.8%)
UICC stage						
0	109 (4.7%)	1576 (16.9%)	474 (10.0%)	48 (1.6%)	971 (7.6%)	3178 (9.9%)
I	570 (24.4%)	4690 (50.3%)	2098 (44.1%)	772 (26.5%)	3932 (30.7%)	12,062 (37.5%)
II	558 (23.9%)	1890 (20.3%)	1396 (29.3%)	1162 (39.8%)	3377 (26.3%)	8383 (26.1%)
III	405 (17.4%)	423 (4.5%)	338 (7.1%)	432 (14.8%)	905 (7.1%)	2503 (7.8%)
IV	423 (18.1%)	86 (0.9%)	96 (2.0%)	167 (5.7%)	672 (5.2%)	1444 (4.5%)
Missing	269 (11.5%)	664 (7.1%)	360 (7.6%)	336 (11.5%)	2970 (23.2%)	4599 (14.3%)
Grading						
1	221 (9.5%)	1852 (19.9%)	820 (17.2%)	237 (8.1%)	1576 (12.3%)	4706 (14.6%)
2	1137 (48.7%)	4796 (51.4%)	2437 (51.2%)	1437 (49.3%)	6211 (48.4%)	16,018 (49.8%)
3 or 4	849 (36.4%)	2240 (24.0%)	1238 (26.0%)	1157 (39.7%)	3405 (26.5%)	8889 (27.6%)
Missing	127 (5.4%)	441 (4.7%)	267 (5.6%)	86 (2.9%)	1635 (12.7%)	2556 (7.9%)
Median follow-up time (years)	7.1	7.8	8.2	8.2	9.9	8.3

**Table 2:** Cox proportional hazard regression models: hazard ratio for all-cause death by the mode of breast cancer diagnosis.

	Simple Model		Multivariable Model ^1^		Simple Lead Time Bias-Corrected Model ^2^		Multivariable Lead Time Bias-Corrected Model ^3^	
Mode of Breast Cancer Detection	HR (95% CI)	*p*-Value	HR (95% CI)	*p*-Value	HR (95% CI)	*p*-Value	HR (95% CI)	*p*-Value
Symptomatic cancer	Ref	-	Ref	-	Ref	-	Ref	-
Mammography screening	0.23 (0.21; 0.25)	<0.001	0.50 (0.45; 0.55)	<0.001	0.36 (0.33; 0.40)	<0.001	0.84 (0.75; 0.94)	0.002
Early detection	0.31 (0.28; 0.34)	<0.001	0.58 (0.52; 0.64)	<0.001	-	-		
Self-examination	0.59 (0.54; 0.65)	<0.001	0.76 (0.68; 0.84)	<0.001	-	-		

^1^ Multivariable Cox proportional hazard regression adjusted for age, UICC stage and grading. ^2^ Simple Cox proportional hazard regression after the correction for lead time bias (with sojourn time by Tabar et al. [24]) showing only mammography screening-detected cancer vs. symptomatic cancer. ^3^ Multivariable Cox proportional hazard regression adjusted for age, UICC, and grading after the correction for lead time bias (with the sojourn time by Tabar et al. [24]), showing only mammography screening-detected cancer vs. symptomatic cancer.

**Table 3:** Cox proportional hazard regression models: hazard ratio for all-cause death by the mode of breast cancer diagnosis for different sojourn times.

	Sojourn Time	Simple Model ^1^		Multivariable Lead Time Bias-Corrected Model ^2^	
Mode of Breast Cancer Detection		HR (95% CI)	*p*-Value	HR (95% CI)	*p*-Value
Symptomatic cancer	-	Ref	-	Ref	-
Mammography screening—no lead-time correction	None	0.23 (0.21; 0.25)	<0.001	0.50 (0.44; 0.56)	<0.001
Mammography screening—lead-time-corrected Tabar	3.7 years (age 50–59) 3.9 years (age 60–69)	0.36 (0.33; 0.40)	<0.001	0.84 (0.75; 0.94)	0.004
Mammography screening—lead-time-corrected Aarts	4.6 years	0.39 (0.35; 0.43)	<0.001	0.90 (0.80; 1.01)	0.061
Mammography screening—lead-time-corrected Choi	2.5 years (age 50–59) 3.1 years (age 60–69)	0.32 (0.29; 0.35)	<0.001	0.73 (0.65; 0.82)	<0.001

^1^ Simple Cox proportional hazard regression only mammography screening-detected cancer vs. symptomatic cancer. ^2^ Multivariable Cox proportional hazard regression adjusted for age, UICC stage, and grading after the correction for lead time bias showing only mammography screening-detected cancer vs. symptomatic cancer.

**Table 4:** Sensitivity analyses: Cox proportional hazard regression models: hazard ratio for all-cause death by the mode of breast cancer diagnosis.

	Multivariable Model ^1^		Simple Lead Time Bias-Corrected Model ^2^		Multivariable Lead Time Bias-Corrected Model ^3^	
Mode of Breast Cancer Detection	HR (95% CI)	*p*-Value	HR (95% CI)	*p*-Value	HR (95% CI)	*p*-Value
Symptomatic cancer	Ref	-	Ref	-	Ref	-
Mammography screening + early detection + self-examination	0.59 (0.54; 0.64)	<0.001	0.40 (0.37; 0.43)	<0.001	0.71 (0.65; 0.77)	<0.001

^1^ Multivariable Cox proportional hazard regression adjusted for age, UICC stage and grading. ^2^ Simple Cox proportional hazard regression after the correction for lead time bias. ^3^ Multivariable Cox proportional hazard regression adjusted for age, UICC stage, and grading after the correction for lead-time bias.

## Data Availability

Due to data privacy restriction, the used dataset cannot be shared freely. However, the provided data set is held by the Cancer Registry of Schleswig-Holstein and can be made available for scientific purposes upon request. Evaluation scripts can be requested from the first author.
